# Therapeutic Intervention with Dietary Chitosan Nanoparticles Alleviates Fish Pathological and Molecular Systemic Inflammatory Responses against Infections

**DOI:** 10.3390/md20070425

**Published:** 2022-06-28

**Authors:** Mona Saleh, Ehab Essawy, Mohamed Shaalan, Shaaban Osman, Fatma Ahmed, Mansour El-Matbouli

**Affiliations:** 1 Clinical Division of Fish Medicine, University of Veterinary Medicine, Veterinärplatz 1, 1210 Vienna, Austria; ehab.essawy@science.helwan.edu.eg (E.E.); mohamedibrahim@cu.edu.eg (M.S.); mansour.el-matbouli@vetmeduni.ac.at (M.E.-M.); 2 Department of Chemistry, Biochemistry Division, Faculty of Science, Helwan University, Cairo 11790, Egypt; 3 Bioinformatics Center, Faculty of Science, Helwan University, Cairo 11790, Egypt; 4 Department of Pathology, Faculty of Veterinary Medicine, Cairo University, Cairo 12613, Egypt; 5 Department of Pharmaceutics and Pharmaceutical Technology, Faculty of Pharmacy, Al-Azhar University, Assiut 71524, Egypt; shaabanosman@azhar.edu.eg; 6 Department of Zoology, Faculty of Science, Sohag University, Sohag 82524, Egypt; 7 Division of Aquatic Animal Health, School of Veterinary Medicine, Badr Universiy, Cairo 11829, Egypt

**Keywords:** chitosan nanoparticles, fish diseases, inflammation, gene expression, pathology, reticulin, rainbow trout, *Yersinia ruckeri*

## Abstract

Marine bio-sourced chitosan nanoparticles (CSNP) are antimicrobial and immunomodulatory agents beneficial for fish medicine. Herein, dietary CSNP was investigated for the amelioration of the systemic inflammatory responses of an induced fish model. One hundred and forty-four rainbow trout were assigned to one pathogen-free and non-supplemented group (negative control), and three challenged groups: non-supplemented (positive control), CSNP-preventive, and CSNP-therapeutic. After a feeding experiment extended for 21 days, the organosomatic indices (OSI) and molecular aspects were assessed. After a challenge experiment extended for further 28 days, CSNP-therapeutic intervention was assessed on fish survival and systemic inflammatory responses on pathology, histo-morphology, and molecular aspects. With CSNP administration, OSI nonsignificantly decreased and the relative expression of targeted inflammatory-mediator genes was significantly increased. The CSNP-therapeutic fish showed an RPS of 80% as compared to the positive control group, and CSNP-therapeutic administration retained the highest gene expression augmentation up to 28 days after the challenge. Notably, the splenic reticulin fibers framework of the CSNP-therapeutic group retained the highest integrity among the groups during the infection. After recovery, reticulin fibers density in the CSNP-therapeutic samples was significantly higher than in the negative control group, which indicates high innate immunity. Thus, CSNP showed promising biotherapeutic features enhancing fish resistance against infections.

## 1. Introduction

Multi-sourced metal nanoparticles have been found to elicit antimicrobial efficacy in vitro [1,2,3], and combat fish diseases in vivo [4,5]. Bio-nanotechnology is a branch of nanotechnology that utilizes several bio-sourced polymeric nanoparticles for a wide range of bio-applications. Recently, several bio-sourced nanoparticles were found to be effective in vitro as antimicrobials against major fish pathogens [6]. Repeatedly, chitosan nanoparticles (CSNP) were reported in biomedical applications in vitro as well as in vivo [7,8]. CSNPs are the nanoform of the marine bio-sourced chitosan polysaccharides and are considered one of the best-known polymeric nanoparticles. They have shown low cytotoxicity and high anti-microbial efficacy, mucoadhesion, and hemocompatibility in several bio-nanotechnological applications in aquaculture [9,10]. CSNPs exhibit antimicrobial effects in vitro against some major pathogenic bacteria and fungi of fish [11,12].

The parent chitosan and CSNP were successfully implicated in fish feed supplementation for immunomodulation and disease resistance [13,14]. Fish feed supplementation with CSNP is considered promising in fish medicine due to its low cytotoxicity and genotoxicity [15,16]. In addition, they enhance fish immunity and improve their survival, growth, and quality [17]. However, further studies are needed to determine the potential systemic immunomodulatory effects of dietary CSNP in fish. Oral administration of CSNP alone or in combination with other biocomponents enhances rainbow trout (*Oncorhynchus mykiss*) health and improves stress tolerance, growth, immunity, and disease resistance [18,19,20]. We have previously demonstrated the efficacy of CSNP as a feed additive for enhancing intestinal immunity and antibacterial defense of *O. mykiss* [21]. That study had suggested that CSNPs are more beneficial in treatment regimens than in the prevention of intestinal infections. Relying on the promising findings of our previous study that showed considerable improvement in the fish disease resistance, the current study has been conducted for further investigations on the efficacy of CSNP administration, in terms of prevention and treatment, in the modulation of *O. mykiss* systemic inflammatory responses during infections. Fish survival, organosomatic indices and morphometry, and pathological and molecular inflammatory responses were assessed. As the H&E-counterstaining is not enough alone for distinguishing the histo-morphometric architecture and the pathologic inflammatory signs during the fish inflammatory responses, we employed a distinctive special histochemical staining for more in-depth pathological investigations. The specific staining technique was applied for the identification of the splenic reticulin fibers framework to elucidate the histo-morphometric architecture and the pathologic inflammatory signs in the lymphoid organs of *O. mykiss*. Reticulin mainly consists of type III collagen, which composes tissue conjunctions, in addition to other types of collagen fibers, glycoproteins, and proteoglycans [22].

Differential reticulin staining mainly detects reticular fibers in basement membranes and is frequently used for the characterization of fish systemic histology for exploring their functional and phylogenetic development. In an earlier study, reticulin staining was used for tracing the phylogenetic development of melano-macrophages centers in fish liver, spleen, and kidney [23]. In addition, reticulin staining was employed as a differential staining method for monitoring the distribution of fish skeletal muscle reticular fibers and collagen during juvenile and adult growth phases [24]. It was also implicated in the characterization and differentiation of fish male and female reproductive phases [25,26,27]. Furthermore, reticular fibers staining was employed for the characterization of the normal gill tissue of rainbow trout [28]. Moreover, reticulin monitoring aids in the exploration of the changes in the gonadal basement membranes of teleost’s sex-reversed females for gonadal remodeling during the sex reversal process [26].

The present study provides an insight into the promising role of CSNP as an immunomodulatory feed additive for fish biotherapeutic applications aiming at enhancing their systemic responses against infections. The main objective of our study is to discover the potential role of CSNP administration in the modulation of inflammatory mediators during infection.

## 2. Results

### 2.1. Physical Characteristics of CSNP

**Figure 1:**
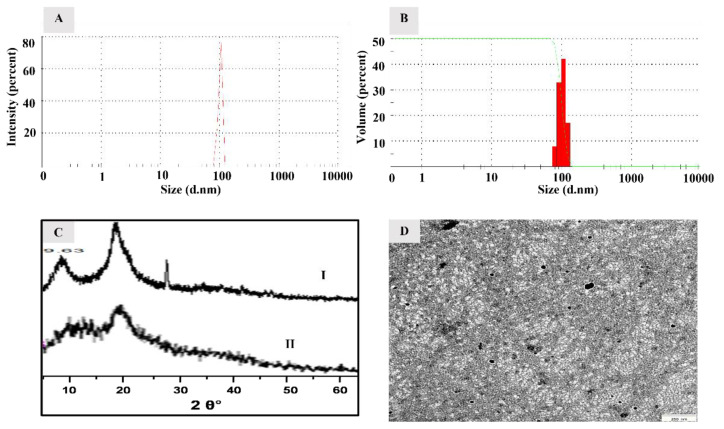
Size and morphology of the modified CSNP. (**A**) Dynamic light scattering peak of CSNP shows an average particle size of 100 nm; (**B**) size distribution pattern shows homogenous (green disciplinary z-shape line) and narrow size distribution range from 73 to 145 nm (red histogram); (**C**) X-ray diffraction pattern of chitosan (I) and CSNP (II) shows the amorphous nano-pattern indicated from the remarkable reduction in the peak intensity, and (**D**) TEM micrograph shows the spherical shape of the particles (black spheres) (scale bar = 250 nm).

Zetasiezer revealed that the mean diameter of the obtained CSNP is 100 nm (Figure 1A), and a narrow homogenous distribution was recorded of the different particles’ sizes (from 73 to 145 nm; Figure 1B). XRD spectra showed a decreased intensity in the characteristic peak of the CSNP crystal lattice (Figure 1CII) compared with the parent chitosan (Figure 1CI), which had indicated an amorphous structure. TEM photomicrographs showed a homogenous distribution of similarly shaped particles having a diameter range from 70.23 to 159.53 nm and an average size of 103.22 nm (Figure 1D).

### 2.2. CSNP Cytotoxicity

**Figure 2:**
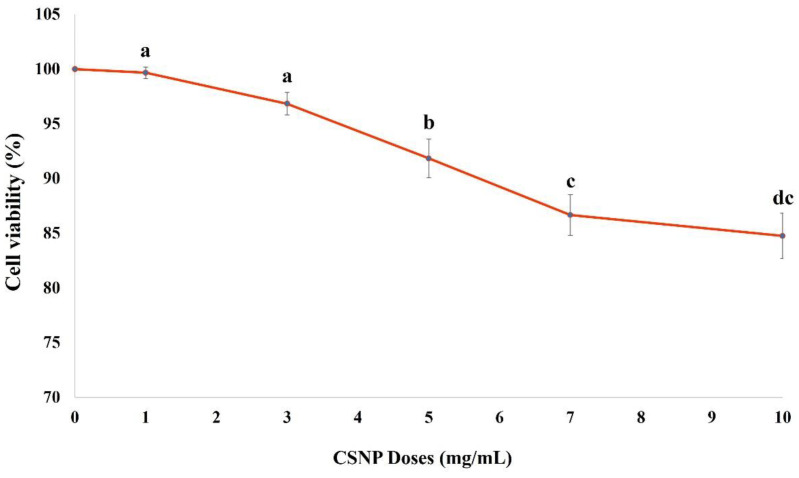
Viability of BF-2 cells assessed by trypan blue exclusion assay after incubation with CSNP for 24 h. The cell viability is expressed as mean percentage ± SD (*n* = 9). No significant differences (*p* = 0.75) were observed in the cell viability with the 1 mg/mL treatment, while significantly lower viability was observed with treatments 3, 5, and 7 mg/mL. Superscript letters indicate the statistical significance difference between doses (*p* < 0.05).

The BF-2 cell viability varied in relation to the CSNP dose, but not with the incubation period. After incubation for 24 h, no significant difference (*p* > 0.05) was observed in the viability of the cells treated with 1 mg/mL compared with the untreated negative control cells. However, at the higher doses of CSNP (3, 5, 7, and 10 mg/mL), cell viability significantly (*p* < 0.005) decreased with an increase in CSNP concentration in a dose-dependent manner in comparison with the untreated cells. All treated cells retained more than 84% of their viability, and no considerable change was observed in the morphology of the treated cells compared with the control cells. Cell viability data after 24 h of incubation are expressed as mean percentages ± SD and displayed in a line chart (Figure 2). Prolongation of the incubation period up to 48 h did not affect cell viability (*p* > 0.05). 

### 2.3. Fish Cumulative Survival Rate and Relative Percent Survival

**Figure 3:**
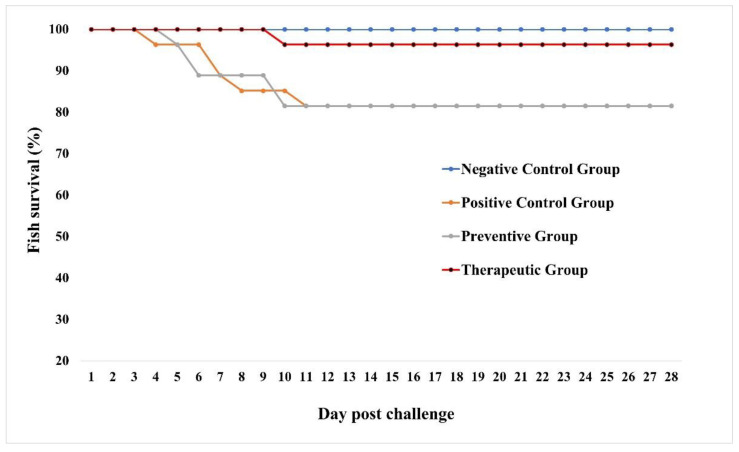
The cumulative survival of *O. mykiss* (CSNP-supplemented or non-supplemented), over 28 days of ERM infection. Sampled fish during the challenge experiment were counted as surviving fish.

Moribund fish were collected occasionally from the ERM-infected tanks during the fish morbidity period starting from the 4th up to 12th day post-challenge (dpc), and they were counted as mortality. The cumulative survival rate of the challenged fish was calculated during the 28 days of the challenge experiment. The survival rate was calculated per day for each replicate tank of each group, considering the sampled fish as survival, [(No. of surviving fish/initial no. of fish) × 100]. The average survival rate (cumulative) of each treatment group was represented in a line graph (Figure 3). 

By the end of the challenge experiment, the relative percent survival (RPS) was calculated for each challenging group (RPS (%) = {[1 − [group mortality (%)/positive control group mortality (%)]} × 100). In comparison with the positive control group, the CSNP-therapeutic group demonstrated 80% RPS, while the CSNP-preventive group demonstrated 0% RPS as it had the same mortality as the positive control group.

### 2.4. Clinical Signs and Systemic Morphometry

**Figure 4:**
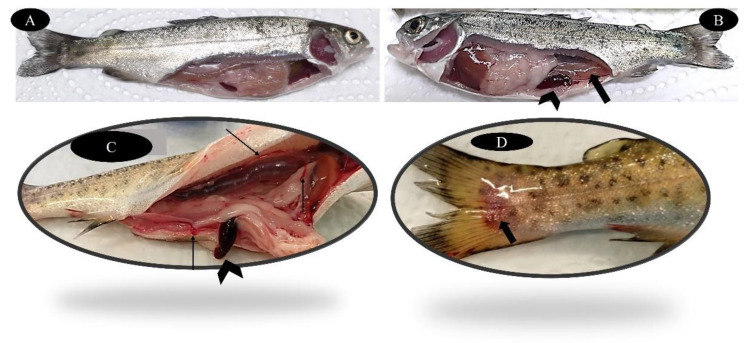
Photomicrograph displays the systemic macroscopic morphometry and clinical signs of ERM-infected *O. mykiss*. (**A**) negative control fish; (**B**) infected fish showing enlarged spleen (chevron); (**C**) splenomegaly (chevron) and systemic inflammatory signs (line arrows), and (**D**) congestions on the fish tail.

Clinical signs associated with the enteric redmouth (ERM) infection include loss of appetite, swimming near tank borders, floating to the water surfaces, dark skin, exophthalmia, and hemorrhagic congestions of the fish tail. During the post-challenge sampling, external inflammations were observed on the lower intestine, liver, and spleen of the challenged fish, and splenomegaly was the most obvious (Figure 4). Mild clinical signs were observed in the CSNP-therapeutic group compared with the other challenged groups. The symptoms of ERM persisted for two successive weeks, and full recovery of behavior and organ morphometry of all groups was reached on the 28th day.

### 2.5. Organosomatic Indices (OSI)

After the feeding experiment, the OSI of the CSNP-treated group decreased with no significant difference (*p* > 0.05) compared with the untreated group. For the CSNP-treated and untreated groups, viscerosomatic indices (VSIs), hepatosomatic indices (HSIs), and spleen-somatic indices (SSIs) recorded 7.48 ± 0.22 and 7.33 ± 0.34, 1.38 ± 0.36 and 1.69 ± 0.29, and 0.85 ± 0.33 and 1.38 ± 0.89, respectively. 

### 2.6. Systemic Inflammatory Responses

#### 2.6.1. Molecular Inflammatory Expressions

**Figure 5:**
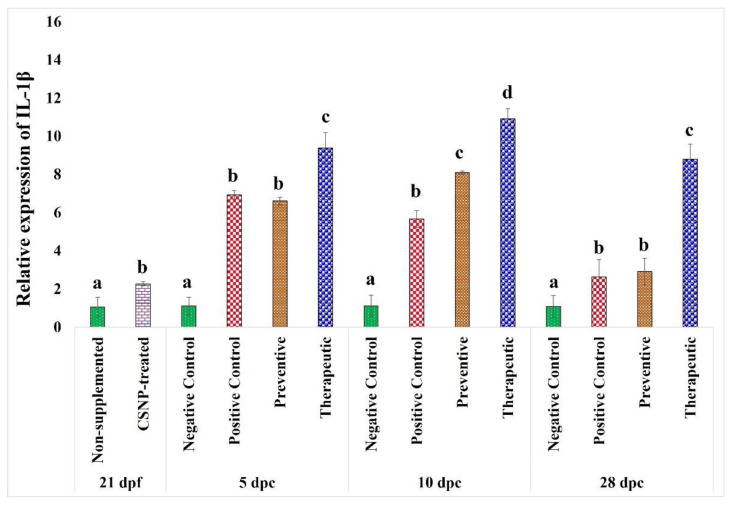
Gene expression of IL-1β relative to the β-actin housekeeping gene in the head kidney of *O. mykiss* (CSNP-supplemented or non-supplemented), before and after challenge. Data are presented as mean ± SD of duplicate technical replicates from triplicate biological replicates per group (*n* = 9). Superscript letters on bars of the same time point indicate the statistical significance difference between groups (*p* < 0.05).

**Figure 6:**
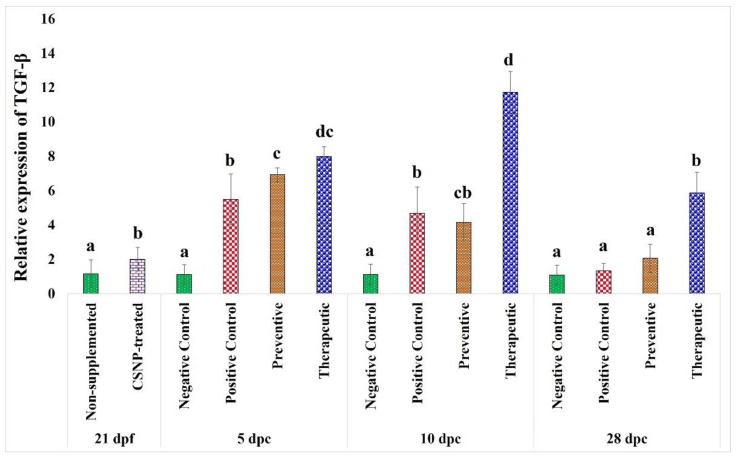
Gene expression of TGF-β relative to the β-actin housekeeping gene in the head kidney of *O. mykiss* (CSNP-supplemented and non-supplemented), before and after challenge. Data are presented as mean ± SD of duplicate technical replicates from triplicate biological replicates per group (*n* = 9). Superscript letters on bars of the same time point indicate the statistical significance difference between groups (*p* < 0.05).

**Figure 7:**
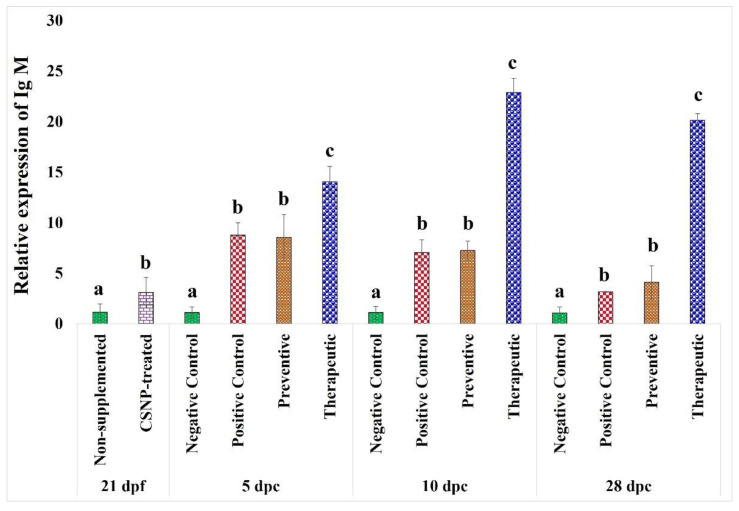
Gene expression of Ig-M relative to the β-actin housekeeping gene in the head kidney of *O. mykiss* (CSNP-supplemented and non-supplemented), before and after challenge. Data are presented as mean ± SD of duplicate technical replicates from triplicate biological replicates per group (*n* = 9). Superscript letters on bars of the same time point indicate the statistical significance difference between groups (*p* < 0.05).

**Figure 8:**
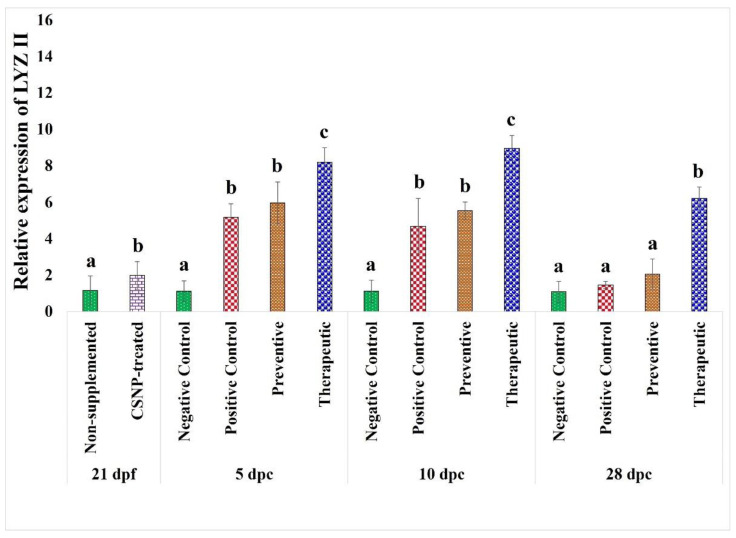
Gene expression of LYZ II relative to the β-actin housekeeping gene in the head kidney of *O. mykiss* (CSNP-supplemented and non-supplemented), before and after challenge. Data are presented as mean ± SD of duplicate technical replicates from triplicate biological replicates per group (*n* = 9). Superscript letters on bars of the same time point indicate the statistical significance difference between groups (*p* < 0.05).

The molecular traits of the head kidney are presented in four-column histograms (Figure 5, Figure 6, Figure 7 and Figure 8) for the relative expression of four inflammatory-mediator genes targeted in our study. On the 21st dpc, the CSNP-treated group showed an increase in the relative expression pattern of the targeted genes compared with that in the untreated group. During ERM infection, the inflammatory-mediator genes of the challenged groups were expressed in different patterns relative to β-actin. At all the time points, CSNP-therapeutic group showed the highest significant augmentation (*p* < 0.05) among all the groups in the relative gene expressions. By contrast, the CSNP-preventive and the positive control groups showed different expression patterns of the targeted genes throughout the sampling time points. On the 5th and 10th dpc, a significant increase (*p* < 0.05) was observed in the expression of all genes in both groups relative to the housekeeping gene as compared with the negative control group. On the 28th dpc, no statistically significant differences (*p* > 0.05) were observed between the groups in the relative expression of their targeted genes. However, the relative expression of both the transforming growth factor-β (TGF-β) and immunoglobulin M (IgM) genes was high (Figure 6 and Figure 7), whereas that of interleukin 1β (IL1-β) and lysozyme II (LYZ II) genes was low (Figure 5 and Figure 8) in both the groups compared with the negative control group.

#### 2.6.2. Histopathological Appraisals

**Figure 9:**
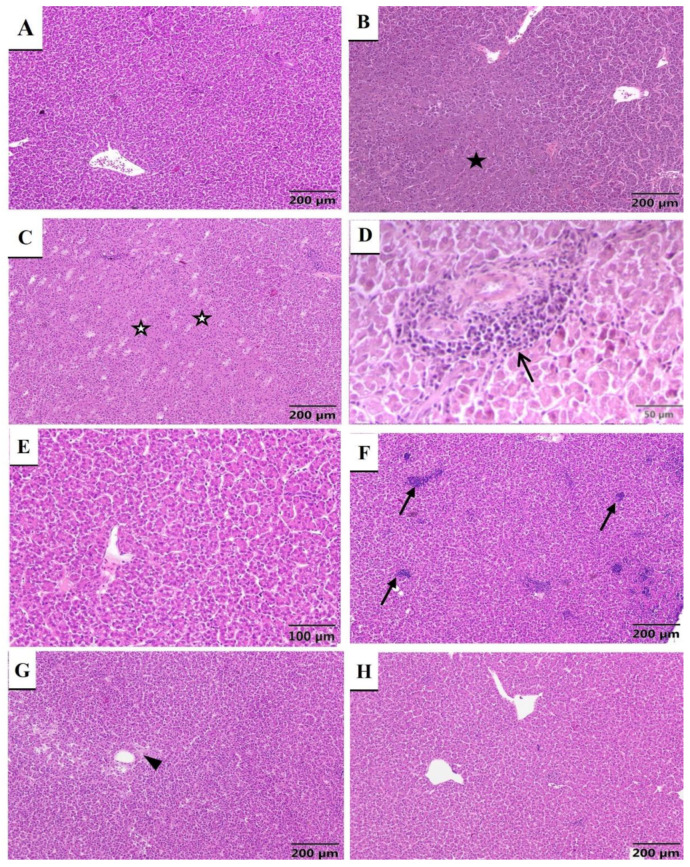
Microphotographs of H&E-stained liver sections of rainbow trout during the challenge experiment; scale bar (50–200 µm). (**A**–**D**) Liver tissues of the experimental groups sampled on the 10th day post-challenge (dpc). (**A**) Negative control group showed normal hepatic histological pattern; (**B**) positive control group showed massive focal area of hepatocytic necrosis with fragmented nuclei and loss of cellular details (asterisk); (**C**) preventive group showed focal areas of hepatic necrosis (white asterisks), and (**D**) therapeutic group showed infiltrations of inflammatory cells (arrows). (**E**–**H**) Liver tissues of the experimental groups sampled on the 28th dpc. (**E**) Negative control group showed normal hepatic histological pattern; (**F**) positive control group showed multifocal aggregates of inflammatory cells in parenchyma (arrows). (**G**) Preventive group showed moderate centrilobular hepatic necrosis with inflammatory cell infiltration (arrowhead). (**H**) Therapeutic group showed normal histological structure.

**Figure 10:**
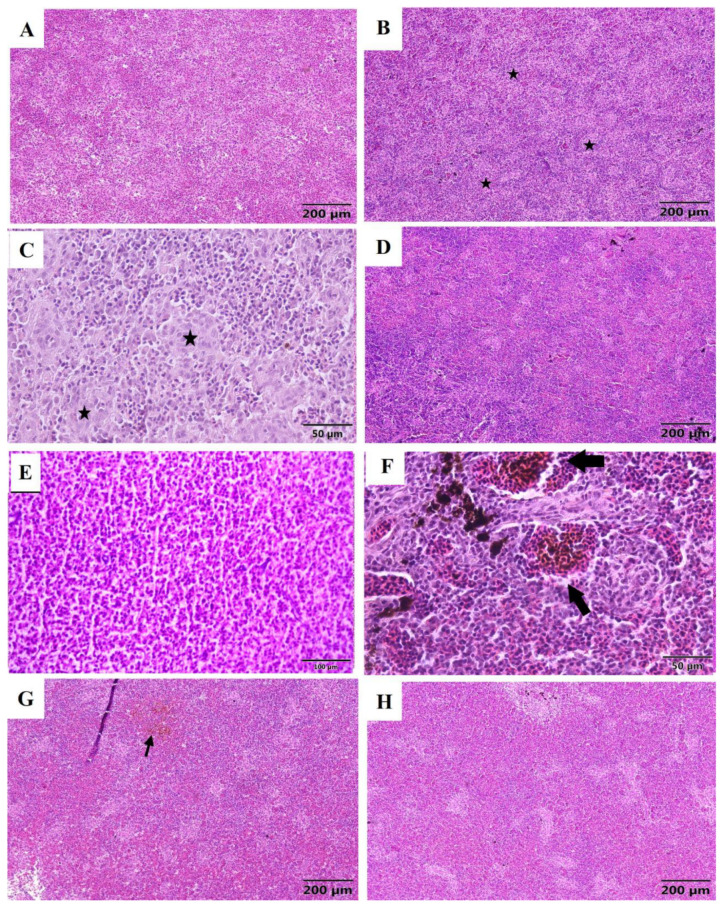
Microphotographs of H&E-stained spleen sections of rainbow trout during the challenge experiment, scale bar (50–200 µm). (**A**–**D**) Spleen tissues of the groups sampled on the 10th day post-challenge (dpc). (**A**) Negative control group showed normal splenic histological pattern; (**B**) positive control group showed moderate depletion of the lymphoid tissue (asterisks); (**C**) preventive group showed moderate depletion of the splenic lymphoid tissue (asterisks), and (**D**) therapeutic group showed normal splenic tissue pattern. (**E**–**H**) Spleen tissues of the experimental groups sampled on the 28thdpc. (**E**) Negative control group showed normal splenic histological pattern; (**F**) positive control group showed severe multifocal hemorrhages and congestion of some blood vessels (arrows); (**G**) preventive group showed mild splenic hemorrhages (arrow), and (**H**) therapeutic group showed normal histological structure.

In the current study, histopathological investigations were conducted on hematoxylin and eosin (H&E) counterstained sections of the liver, head kidney, and spleen. The CSNP-therapeutic group had the least signs of pathological lesions among the challenged groups. Systemic inflammatory signs were detected in different patterns on the liver and spleen of the positive control and CSNP-preventive groups at all time points, except for the 5th dpc, where no obvious differences between the groups were observed. On the 10th dpc, few lesions were observed on the investigated organs of the challenged groups. The positive control group showed massive focal hepatocytic necrosis associated with nuclei fragmentation and cellular details loss (Figure 9B). In addition, moderate depletion of the splenic lymphoid tissue was observed (Figure 10B). Focal hepatic necrosis and moderate splenic lymphoid depletion were occasionally associated with tissue congestions particularly around blood vessels in the CSNP-preventive group (Figure 9C and Figure 10C). Infiltrations of inflammatory cells were observed in the hepatic portal areas of the CSNP-therapeutic group (Figure 9D).

On the 28th dpc, severe lesions were observed on the investigated organs of the challenged groups, except for the CSNP-therapeutic group, which showed the normal pathological pattern. The hepatic parenchyma of the positive control group had multifocal aggregates of inflammatory cells (Figure 9F). In addition, severe splenic multifocal hemorrhages associated with blood vessel congestions were observed (Figure 10F). The CSNP-preventive group showed moderate necrosis and inflammatory cell infiltration in the hepatic central lobules in addition to some mild splenic hemorrhages (Figure 9G and Figure 10G). Regarding the head kidney pathology, no obvious differences were observed between the groups at any of the examined time points.

#### 2.6.3. Splenic Histomorphometry

**Figure 11:**
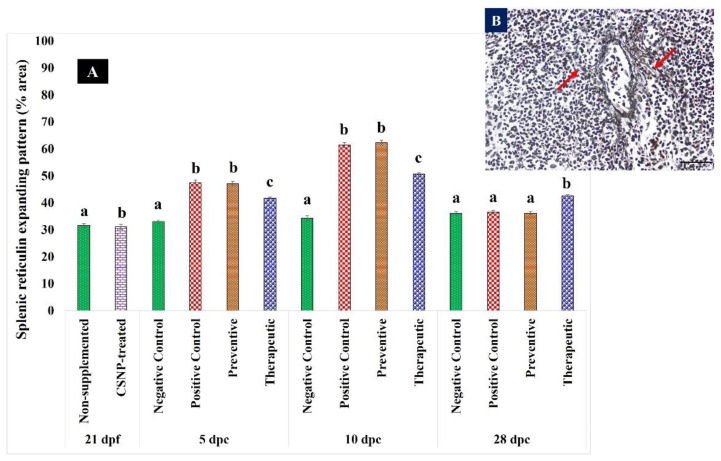
Splenic reticulin expanding pattern (% area) in three arbitrary Gömöri-stained sections per fish. (**A**) Displaying the data as mean values ± SD of 9 sections per group (*n* = 9) in a bar histogram; superscript letters on bars of the same time point indicate the statistical significance difference between groups (*p* < 0.05), and (**B**) representative photomicrograph (40×) shows the normal splenic reticulin fibers (arrows); scale bars (50 µm).

**Figure 12:**
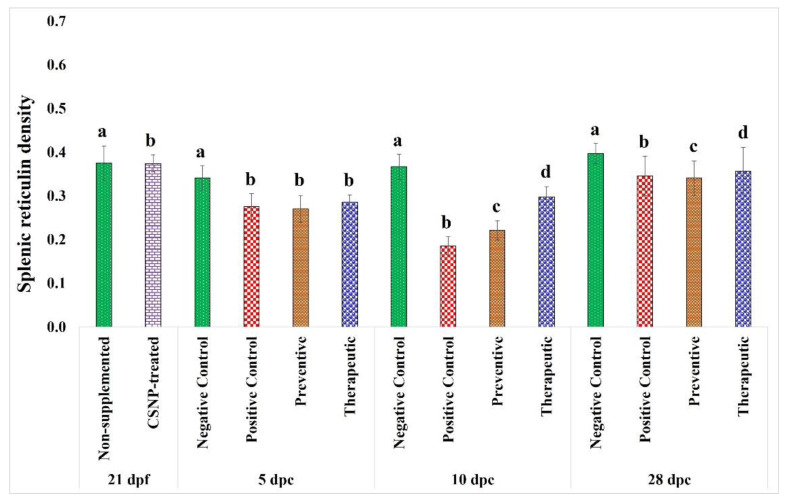
Splenic reticulin density per 0.5 mm^2^ area of each arbitrary Gömöri-stained section per fish. The data are represented as mean values ± SD of 9 sections per group (*n*= 9) in a bar histogram; superscript letters on bars of the same time point indicate the statistical significance difference between groups (*p* < 0.05).

The splenic histomorphometry was evaluated by histochemical staining for the quantitative differentiation of the reticulin fibers in its framework. This differentiation is presented in two parameters: The expanding pattern of these fibers in the total area of the examined photos (% area) (Figure 11), and the density of these fibers per 0.5 mm^2^ area (Figure 12). On the 21st day post-feeding (dpf), no significant differences were observed between the groups in terms of their splenic reticulin density or expanding pattern (% area). During the infection, the reticulin staining density of the spleen from the challenged groups was significantly (*p* < 0.05) lower, whereas the expanding pattern of fibers was significantly higher (*p* < 0.05) compared with the negative control group. The CSNP-therapeutic group showed the highest density and the lowest expansion of the reticulin among the challenged groups, but not the negative control, which kept the highest reticulin density and the lowest expansion of all groups. Notably, on the 28th dpc, the CSNP-therapeutic group recorded a significantly (*p* < 0.05) high expanding pattern of reticulin fibers, with no statistically significant difference (*p* > 0.05) in the reticulin density compared with the negative control group. 

## 3. Discussion

The current study showed that the CSNP treatment considerably ameliorates the pathological and molecular inflammatory responses of ERM-infected rainbow trout. These outcomes support our previous findings, which indicate that dietary CSNPs increase fish antibacterial defense and intestinal immunity, suggesting their promising role in biotherapeutic applications for the benefit of the aquaculture industry [21]. In the current study, no significant difference was recorded in OSI, and no obvious difference was observed in the morphometry and histology of the lymphoid organs of the CSNP-supplemented group compared with the non-supplemented group. However, significant enhancement was observed in the expression pattern of the inflammatory-relevant genes of the CSNP-treated head kidney, which indicates that dietary CSNP increases fish immunity.

In fish, the head kidney is the primary hematopoietic organ, whereas the spleen is a secondary hematopoietic organ [29]. Therefore, we conducted the gene transcription analysis on the head kidney for monitoring *O. mykiss* immunomodulation. Four inflammatory-mediator genes were targeted during our study, namely IL1-β (a proinflammatory cytokine gene), TGF-β (an anti-inflammatory cytokine gene), LYZ II encoding gene (implicated in the production of lysozyme protein), and Ig M encoding gene (involved in antibody production). CSNP-treatment increased the expression of all the targeted genes in different patterns. Furthermore, the CSNP-therapeutic group showed the highest upregulation of all the targeted genes among the groups during ERM infection. IL-1 β induces cytokine production, and TGF-β induces early inflammatory reactions against the infections. Lysozyme protein is involved in the nonspecific innate immune responses against infections, whereas Ig M is involved in adaptive immunity and cytokine production for antibacterial defenses against invading extracellular pathogens. This clarifies, on transcriptomic levels, the high efficacy of therapeutic intervention with CSNP in fish immunomodulation.

During the first two weeks of the infection, the challenged groups showed excess mucus secretions as a defense strategy for pathogen elevation during the host-pathogen interaction [30]. Several clinical signs were observed in the challenged fish; notably, the CSNP-therapeutic group showed the mildest signs of discomfort and left the highest cumulative survival rate, which indicates that this group had the highest resistance among all the groups. Throughout our study, fish systemic inflammatory responses during ERM infection were investigated pathologically on three organs, the head kidney, liver, and spleen, molecularly on the head kidney, and histomorphometrically on the spleen. Histopathological investigations revealed that the positive control and CSNP-preventive groups elicited increased inflammatory lesions during the infection. By contrast, the CSNP-therapeutic group showed almost the normal pathological pattern of all the investigated organs. This indicates high innate immunity of the therapeutic group, which is affirmed by the upregulation of the expression levels of the inflammatory-mediator genes TGF-β and Ig M. The tissue patterns were visible on H&E-counterstained sections; however, the H&E staining cannot distinguish the fibrous texture of the lymphoid tissues, which reflects inflammatory reactions. Therefore, reticulin staining was applied in the current study to distinguish the reticular fiber crosslinks, which form a fine background meshwork holding the cells and supporting the lymphoid organs [31], for the diagnosis of the sinusoidal architecture integrity during ERM infection, which in turn clarifies the anti-inflammatory efficacy of CSNP administration. The spleen was selected for our histomorphometry investigations as splenomegaly was observed to be the most obvious clinical and pathological sign.

After the challenge, the splenic tissues of the 5th and 10th dpc samples from all the challenged groups showed a significant decrease in reticulin density and a significant increase in its expanded pattern compared with the control group. However, the reticulin of the CSNP-therapeutic group showed a significantly higher density and lower area than all other challenged groups. However, no significant differences were observed between the positive control and CSNP-preventive groups in terms of their reticulin fibers density or area. This indicates that the therapeutic group had mildly inflamed tissues compared with the other groups. On the 28th dpc, the spleen of all the challenged groups showed considerable sinusoidal architecture integrity, and significantly high integrity was observed in the therapeutic group, which recorded the highest density and the lowest expanding pattern of reticulin fibers among the challenged groups and was closer to the control. Notably, after 49 days of feeding with CSNP, a complete histopathological recovery associated with a significantly higher expanding pattern of the splenic reticulin was observed in the CSNP-therapeutic group than in the untreated non-challenged control samples. This might be because the teleost spleen is an antigen-trapping organ, which traps antigens within the reticular framework of its white pulp as an innate immune defense [32]. Our results indicate that the gained immunity of the CSNP-therapeutic group enhanced the spleen functions of trapping *Yersinia ruckeri* antigens during the infection for extracellular localization. This is in line with our molecular investigations that showed significant upregulation in the expression of immune-relevant genes, Ig M and LYZ II, in the kidney of the CSNP-treated fish compared with the negative control group. In agreement with our findings, Secombes and Manning [32] reported that the common carp immunized with the cellular bacterial antigens of *Aeromonas salmonicida* showed higher antigen trapping capability and higher expedition of the reticular fibers in the splenic white pulp than did unimmunized fish, which did not receive antigens.

In a similar context to our study, Wahli et al. [33] reported that the dermal reticulin fibers in vitamin C-treated rainbow trout were enriched after 21 days of treatment until they matched the control group. This indicates the tissue regeneration and repair aspects of vitamin C intake during the wound healing process, which starts with the formation of new epidermal cells to close the wound [34]. This process, in turn, requires the reticulin framework holding the newly formed cells to increase; therefore, the reticulin staining density of the tissues were enriched.

A higher staining density of intact tissue from an unimmunized animal than that of the control indicates the over-formation of reticular fibers. This refers to a marked structural change in the organ and is a pathological sign of an immune deficiency disorder that causes severe inflammatory reactions, leading to a high proliferation of the enlarged inflammatory cells associated with multifocal branching of the basement membranes [35]. Therefore, reticular fibers staining was implicated in the diagnostic and therapeutic applications of fish disease. It was contributary in screening the ultrastructure of fish liver tumor and applied for diagnosing the type of a smooth muscle tumor on the fish head [36,37]. Hobbie et al. [35] employed reticulin staining for distinguishing between neoplastic and nonneoplastic fish liver lesions, which is the same lesion type observed in our study.

## 4. Materials and Methods

### 4.1. Supplemented Diet Preparation

**Scheme 1:**
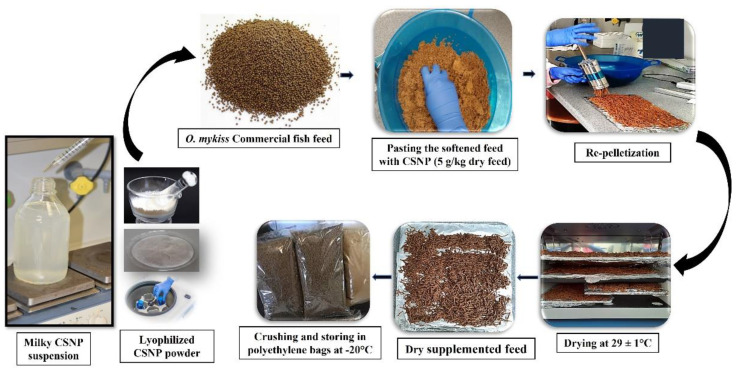
Representative scheme of the preparation of the supplemented feed performed in our labs. Preparation of CSNP was performed by the ionic gelation method followed by incorporation of 5 g/kg of dry fish feed.

A supplemented diet was prepared as described in detail in our previous study [21]. Briefly, CSNPs were prepared via the ionic gelation of an acidic solution (0.5% *w*/*v* in dilute aqueous acetic acid (1%, *v*/*v*)) of low molecular weight chitosan (50 kDa, 90% deacetylation degree) with the dropwise addition of 1:3 *v*/*v* of an aqueous solution of sodium tripolyphosphate salt (0.25%, Sigma-Aldrich, Vienna, Austria). The reaction was conducted after adjusting the pH of chitosan solution to 4.6–4.8 by using NaOH (10 N) [38]. The obtained milky CSNP suspension was centrifuged at 4000× *g* for 30 min at 4 °C, lyophilized, powdered, and stored at −20 °C for further use. A high/safe dose of powdered CSNP (5 g/kg dry feed weight) was kneaded with *O. mykiss* commercial feed (Aqua Dynamic Semi Swim/2, Garant Aqua) after softening in minimum amount of distilled water to prepare the supplemented feed. The obtained stiff dough of the supplemented feed was re-pelletized via pressing in 1 mm holes of a manual extruder machine, fully dried at 29 ± 1 °C, and stored at −20 °C in sealed polypropylene bags until further use [13,39] (Scheme 1).

### 4.2. Physical Characteristics of the Prepared CSNP

The physical and cytotoxic characteristics of the obtained CSNP were assessed. The physical characteristics, including the shape and size of the particles, and the powder pattern were considered as reported in our previous studies [11,21]. A scanning electron microscope (SEM; Phillips-500, Hamburg, Germany) was used for detecting particle morphology. A transmission electron microscopy (TEM; EM900, Zeiss, Oberkochen, Germany) was used for detecting the particles’ shape and average diameter. A Zetasizer (Nano ZS^®^) of dynamic light scattering was used for estimating the average size and the size distribution of particles. An X-ray diffractometer (PW 1729, Philips, Eindhoven, The Netherlands) was used for the detection of the X-ray diffraction (XRD) pattern of the obtained CSNP powder.

### 4.3. Cytotoxic Characteristics of the Prepared CSNP

CSNP cytotoxicity was investigated in vitro on the fry host cell line, Fibroblastoid Bluegill fry Lepomis macrochirus (BF-2) following trypan blue exclusion assay in accordance with the method reported by Qi et al. [40] with minor modifications. In brief, a minimal essential medium (MEM, Gibco, Waltham, MA, USA) containing 2% fetal bovine serum was specified for cell cultivation on a 24-wells tissue culture plate. In two 24-wells plates, 100 µL of MEM/BF-2 containing approximately 2 × 105 cells/cm^2^ was seeded into each triplicate well containing 1 mL pure MEM. Then the culture plates were incubated at 20 °C for 24 h to allow cell adhesion until confluent monolayer was formed. Accurately weighed (Xs Balance mod. 224–220 gr. −0.1 mg) CSNP powder was mixed with MEM to obtain serial doses of 1, 3, 5, 7, or 10 mg/mL. The obtained CSNP-MEM suspensions replaced the media in the replicate wells, except for an additional single untreated negative control well per plate. The plates were further incubated for 24 h or 48 h under the aforementioned conditions. Subsequently, the culture media were removed, the residual CSNP was carefully washed using phosphate-buffered saline (PBS), and trypan blue staining was performed. Equal volumes of trypan blue dye (0.4%, Sigma-Aldrich, Vienna, Austria) and PBS containing washed cells were mixed in each well for 1 min. Thereafter, the dye–PBS mixture was replaced by 4% formalin and left for 10 min for fixation. The cells were rinsed thrice in PBS and were examined under an inverted microscope for their viability. The number of unstained cells (viable and intact) versus the number of blue-stained (disrupted) cells was estimated in triplicate 100 cells per well, (i.e., three fields of 100 cells each). The percentage of viable cells in each well was calculated, and cell viability (cell survival) was expressed as mean percent ± standard deviation (SD) (*n* = 9).

### 4.4. Fish Rearing and the Experimental Design

The current study was conducted at the experimental facilities of Department for Farm Animals and Veterinary Public Health, Clinical Division of Fish Medicine, University of Veterinary Medicine, Austria after obtaining approval from the Ministry of Science, Austria considering the section§26ff of the Austrian laws for the care and use of experimental animals (GZ: 2020-0.001.578). The guidelines of the European institutional ethics and animal welfare were followed for fish care and management.

*O. mykiss*, bought from a certified fish farm, were transported to our wet laboratory and acclimated for 14 days. The obtained fish had an average total body weight of 15.53 ± 0.67 g and an average total body length of 11.06 ± 0.46 cm (means ± SD). For fish harboring, 75 L tanks with a flow-through system (0.5 L/s), adequate aeration (9 ± 0.5 ppm dissolved oxygen), and electric rod heaters were used. Fish were fed twice daily with commercial feed (Aqua Dynamic Semi Swim/2, Garant Aqua, Vienna, Austria) ad libitum and kept in 12 h light-dark conditions. The water quality parameters of pH, temperature, and dissolved oxygen were checked daily. The levels of nitrate, nitrite, carbonate, and total hardness were surveyed once per week.

**Scheme 2:**
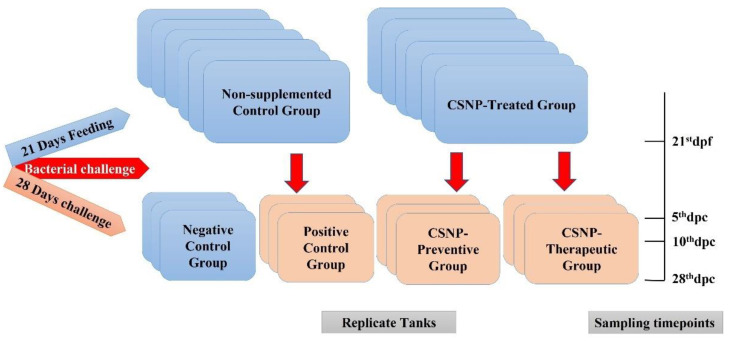
The experimental design and sampling time points during the current study. For 21 days feeding, two initial feeding groups were included: CSNP-treated and untreated control. The challenge trial was initiated by exposing *O. mykiss* to live *Y. ruckeri* (CSF007-82) for 2 h. Four experimental groups were considered including negative and positive controls and CSNP-prevented and therapeutic groups. The fish were sampled at four time points, including the 21st dpf, and the 5th, 10th, and 28th dpc.

The experiment was conducted for 49 days on 144 equal-sized, healthy acclimated *O. mykiss*. The fish were randomly selected and equally distributed into twelve 75 L indoor fiberglass tanks, with a primary stocking density of 12 fish per tank, comprising four experimental groups in triplicate. The experimental groups were subjected to feeding experiment spanning 21 days followed by a challenge experiment spanning 28 days (Scheme 2), and considering the ”tank effects”, all tanks were exposed to as similar conditions as possible. The fish were fed twice daily ad libitum. Two groups received commercial feed supplemented with CSNP (5 g/kg dry feed) and served as CSNP-treatment groups, and two groups received non-supplemented commercial feed and served as positive or negative untreated control groups. The CSNP dose was determined according to the promising findings of several previous studies [14,41]. During our study, the approximate feed consumption by fish was 2% of their body weight daily, (i.e., 0.3106 g); therefore, the approximate CSNP dose was reached 1553 mg/individual fish, (i.e., 0.1 mg/g of fish body weight).

After 21 days of feeding, the first fish sampling was conducted; then, the remaining fish, except for the negative control group, were challenged with *Y. ruckeri* (2 × 10^6^ CFU/mL of water), the causative agent of ERM disease of salmonids. All the groups continued to receive the same feed regimen during the experiment, except for one of the treatment groups, which received non-supplemented feed in replacement of the supplemented feed throughout the challenge experiment. Thus, the treatment groups consisted of CSNP-preventive or CSNP-therapeutic groups [33]. The experiment lasted for 49 days, during which wastes, or feed leftover were siphoned from the tanks 1 h after each feeding time to maintain cleanliness.

### 4.5. Fish Health/Disease Status

The pathogen-free status of the fish was confirmed upon the fish arrival from the fish farm. The infection status was confirmed after the bacterial challenge. Fecal contents and the brain, kidney, and spleen of the sampled or moribund fish were spread and inoculated on blood agar plates (Sigma-Aldrich, Vienna, Austria). The agar plates were incubated for 10 days at 20 °C and were frequently observed for bacterial growth [4].

### 4.6. Bacterial Challenge

On the 21st day after feeding with CSNP, the remaining fish of all the experimental groups were exposed to 2 h bath immersion with the virulent isolate of *Y. ruckeri* (CSF007-82) for the experimental challenge, except for the untreated negative control group, which was exposed to sterile PBS (Sigma-Aldrich, Vienna, Austria). The bacterial isolate was obtained from the National Centre for Cool and Cold-Water Aquaculture, Kearneysville, WV, USA, and stored in the Clinical Division of Fish Medicine, University of Veterinary Medicine, Vienna at −80 °C. Before use, tryptic soy broth (Sigma-Aldrich, Vienna, Austria) was inoculated with one colony of the stored *Y. ruckeri* and was cultured overnight at 22 °C in an orbital incubator at 144 revolutions per minute. The experimental challenge was performed through bath exposure to a theoretical concentration of 2 × 10^9^ CFU ml^−1^ from the bacterial suspension (assessed using a spectrophotometer, Eppendorf BioPhotometer^®^, Eppendorf, Hamburg, Germany) for 2 h while lowering the water level to 50 L/tank to reach a final concentration of 2 × 10^6^ CFU ml^−1^ [42]. During the challenge, the water flow was temporarily stopped and resumed after 2 h of exposure, whereas the airflow was constantly maintained to spread the infection while preventing fish mortality. The challenge trial was continued for further 28 days, during which four sampling time points were considered.

### 4.7. Fish Sampling and Tissue Collection

Fish were sampled once before the challenge experiment, on the 21st dpf, for the organosomatic indices estimation, and at three sampling time points during the challenge experiment, namely on the 5th, 10th, and 28th days after the challenge for the investigations of fish systemic inflammatory responses. The fish were starved for 24 h before sampling to ensure the postabsorptive state of fish, and one fish was collected from each replicate tank, (i.e., three fish/group) for dissection. An overdose (300 mg L^−1^) of buffered tricaine methane sulfonate (MS-222) was used for fish euthanasia. On the 21st dpf, the liver and whole viscera exclusive of liver were excised and weighed individually (Xs Balance mod. 224–220gr. −0.1 mg) for the assessment of the organosomatic indices relative to fish average body weight. The liver, head kidney, and spleen were excised from each dissected fish and histomorphology examination was conducted. Small pieces of the excised liver and spleen were fixed in 10% neutral buffered formalin for histological and pathological investigations. Pieces of the head kidney were kept in RNA later™ (Sigma-Aldrich, Vienna, Austria) at 4 °C for the gene expression analysis.

### 4.8. Clinical Signs and Fish Cumulative Survival

The challenged fish were closely monitored thrice a day for the identification of clinical signs characterizing the ERM infection. Moribund fish were removed from the tanks immediately on detection and euthanized humanely by using an MS-222 overdose. For fish survival assessment, morbidity was recorded as the mortality count. Fish cumulative survival was considered in addition to the calculation of RPS in accordance with a study by Amend [43] and Saeidi asl et al. [44] by using Equation (1).

RPS (%) = {[1 − [group mortality (%)/positive control group mortality (%)]} × 100(1)

### 4.9. Organosomatic Indices and Systemic Morphometry

VSI, HSI, and SSI were calculated after feeding the fish with CSNP for 21 days from the average weight of the excised whole viscera, liver, and spleen relative to the average body weight of the sampled fish according to the equations (2, 3, and 4) as described by El Basuini et al. [45]. The obtained data were expressed as means ± SD (*n* = 9/treatment).

VSI (%) = Viscera weight (g) × 100/ Body weight (g)(2)

HSI (%) = Liver weight (g) × 100/ Body weight (g)(3)

SSI (%) = Spleen weight (g) × 100/ Body weight (g)(4)

During dissection, the morphometry of all the internal organs was observed for any visible systemic inflammatory signs during ERM infection.

### 4.10. Systemic Inflammatory Response Investigations

#### 4.10.1. Gene Transcription Analysis

The inflammatory response of *O. mykiss* was investigated based on the expression pattern of four immune-related genes in the head kidney. Two inflammatory mediator genes were selected, namely pro-inflammatory IL1-β and the anti-inflammatory TGF-β. Two innate immune mediator encoding genes for antibody and lysozyme production were selected, the lysozyme II (LYZ II), and the immunoglobulin M (Ig M) heavy chain. We aimed to investigate, on a transcriptional level, the modulatory effect of dietary CSNP on the systemic immunity of rainbow trout during *Y. ruckeri* infestation.

- Extraction of Total RNA and Complementary DNA Synthesis

Total RNA was extracted routinely for the relative messenger RNA (mRNA) expression analysis. RNeasy Mini Kit (Qiagen, Hilden, Germany) manual, including on-column DNase I digestion step (Qiagen, Hilden, Germany), was followed for the extraction of pure mRNA. Qualification and quantification of the extracted RNA were performed using a NanoDrop 2000 spectrophotometer (Thermo Fisher Scientific, Vienna, Austria). Thereafter, complementary DNA (cDNA) was synthesized from a fixed input of mRNA (1 µg) according to the manual of the iScript cDNA synthesis kit (Bio-Rad, Hercules, Munich, Germany) users [46].

- Reverse Transcriptase Quantitative Real-Time PCR (RT-qPCR)

During the current RT-qPCR analysis, β-actin was selected and assigned as the housekeeping gene normalizing the expression of targeted genes [47,48]. The CFX96 Touch Real-Time PCR detection system (Bio-Rad, Munich, Germany) and qPCR SYBR Green Supermix were used for the assay in this study. Primer sequences, accession numbers, and the amplicon sizes of genes targeted in the current analysis are listed in Table 1. 

The qPCR analysis was conducted on a final volume of 10 µL, which contained 1 µL of cDNA (diluted 5 folds), 5 µL SYBR Green Supermix (5x Sso Advanced™ Universal, Bio-Rad, Hercules, CA, USA), 3 µL DNA-free water (Bio-Rad), and 0.5 µL of primer dilution 5 pmol/µL. The cycling setup of the RT-qPCR machine for cDNA amplification consisted of four successive steps: initial cDNA denaturation (1 cycle at 95 °C for 5 min), final cDNA denaturation (45 cycles at 95 °C for15 s), primer annealing (1 cycle at 55 °C for 15 s), and finally cDNA extension (1 cycle at 72 °C for 15 s). This setup ensured the formation of double-stranded cDNA. At the end of each cycle, the melting curve detection of non-specific primer binding was obtained by gradually increasing the temperature by 0.5 °C every 10 s, starting from 55 °C up to 95 °C. The fold change between the experimental groups in terms of the relative expression of mRNA was determined at each sampling time point based on the 2^−∆∆Ct^ method. CFX Maestro Software (Bio-Rad Laboratories Inc., Munich, Germany) was used for the expressions’ normalization assessment. The performance of the RT-qPCR analysis for each gene was controlled using duplicate template and duplicate transcriptase controls [52,53].

#### 4.10.2. Pathology and Histomorphology

Formalin-fixed tissue pieces of the liver and spleen were processed to obtain paraffin-embedded blocks, and then ultrathin sections (4 µm) were prepared by using an HM 360^®^microtome. For pathological investigations, triplicate sections of each organ per group were counterstained with H&E [54] and were examined carefully under a light microscope (Olympus BX 46). Lesions observed were photographed using an Olympus DP 21 digital camera (Olympus Corporation, Tokyo, Japan) connected with the used microscope. The spleen was subjected to microscopic histomorphological examinations by using the differential histochemical staining technique of Gömöri’s silver impregnation, particularly for reticulin fibers differentiation [55]. Splenic reticulin fibers were quantitatively measured on three out sections of every five successive Gömöri-stained sections per group by using ImageJ software [56].

### 4.11. Histomorphometry and Statistical Analysis

ImageJ software (version 1.41o, Public Domain, BSD-2, https://imagej.net/Ops) was used for the quantitative measurements of splenic reticulin fibers according to the method reported by Eliades et al. [56]. Randomly chosen gridded digital images (acquired at 40×) were selected in triplicate for the estimation of the number of reticular fiber-to-fiber bands in an area of 0.5 mm^2^ area for each image from the triplicate sections of each group, (i.e., *n* = 9).

For the statistical analysis, all data were obtained from nine independent replicates, which were expressed as means ± SD. The data were checked for normality and were analyzed statistically by using the statistical package for social sciences (SPSS) software, version 25 (IBM Corporation, Armonk, NY, USA). The groups were compared using the analysis of variance (ANOVA), and the paired comparison of means was performed using Duncan’s post hoc test for the detection of significant differences between groups. The data probability value *p* < 0.05 at > 95% confidence level refers to the statistical significance.

## 5. Conclusions

In the present study, the systemic immunomodulation of CSNP-treated *O. mykiss* and their inflammatory response during the ERM infection were investigated. CSNP-administration induced the expression of all the targeted inflammatory-mediator genes in different patterns. Therapeutic intervention with CSNP during the ERM infection revealed mild signs of discomfort and decreased fish morbidity. In addition, increased molecular expressions, mild pathological lesions, and a relatively unaffected splenic reticulin framework were observed. These findings indicate that the therapeutic administration of CSNP by *O. mykiss* increases their resistance and inflammatory responses to infections.

## Figures and Tables

**Table 1:** List of primers used for the qRT-PCR gene expression analysis.

Gene	Primer Sequence (5′–3′)	Gene Bank Accession No.	Amplicon Size (bp)	Reference
β-actin	F: ATGGAAGGTGAAATCGCC	AF157514	260	[49]
	R: TGCCAGATCTTCTCCATG			
IL-1β	F: TATCCCATCACCCCATCACC	AJ223954	350	[21]
	R: GGCGTGACGTACGAAGACAGG			
TGF-β	F: AGTTGCCTTGTGATTGTGGG	X99303	404	
	R: GGGGTGGGCAGAGGCTCCGG			
LYZ II	F: GCTGTTGTTCTCCTGCT	NM_001124716.1	129	[50]
	R: GCAAACCCAGTTGGGCAG			
Ig M	F: ACTGCTCCGACTTTGTTCCC	XM_036941450.1	160	[51]
	R: CCGCAGGGTACTGAACGAAA			

## Data Availability

All data are contained within the article.
